# Right ventricular remodeling after conduit replacement in patients with corrected tetralogy of Fallot - evaluation by cardiac magnetic resonance

**DOI:** 10.1186/s13019-019-0899-6

**Published:** 2019-04-15

**Authors:** Henrik Guné, Johan Sjögren, Marcus Carlsson, Ronny Gustafsson, Pia Sjöberg, Shahab Nozohoor

**Affiliations:** 1 Department of Cardiothoracic Surgery, Clinical sciences, Lund University, Skane University Hospital, SE-221 85 Lund, Sweden; 2 Department of Clinical Physiology, Clinical sciences, Lund University, Skane University Hospital, Lund, Sweden

**Keywords:** Adult congenital heart disease, Pulmonary valve replacement, Outcome, Right ventricular function

## Abstract

**Purpose:**

To evaluate the potential for right ventricular reverse remodelling after pulmonary valve replacement using cardiac magnetic resonance imaging, in adults with corrected tetralogy of Fallot and severe pulmonary insufficiency.

**Material and methods:**

Ten patients with previous correction of tetralogy of Fallot with severe pulmonary insufficiency accepted for pulmonary valve replacement were evaluated prospectively with cardiac magnetic resonance imaging preoperatively and re-evaluated 10 ± 5 months postoperatively. Follow up for survival was 100% complete with mean of 37 ± 12 months.

**Results:**

The preoperative mean indexed right ventricular end-diastolic volume was reduced from 161 ± 33 ml/m^2^ to 120 ± 23 ml/m^2^ postoperatively, *p* < 0.001. The preoperative mean indexed right ventricular stroke volume was reduced from 72 ± 20 ml/m^2^ to 50 ± 6 ml/m^2^ postoperatively, *p* = 0.002. After pulmonary valve replacement, the right ventricular ejection fraction did not change significantly (46% versus 42%, *p* = 0.337). Pulmonary insufficiency fraction decreased from 49% ± 11 to 1% ± 1 postoperatively, *p* < 0.001.

**Conclusions:**

Pulmonary valve replacement leads to a favourable early reverse remodelling with a reduction in RV volumes and improved function in all patients regardless of their preoperative indexed right ventricular volume.

## Introduction

Tetralogy of Fallot (TOF) is one of the most common forms of cyanotic congenital heart disease. Patients with TOF undergo cardiac surgery in the early years of childhood. Surgical correction is performed with excellent results and early mortality is low [1]. However, cardiac interventions in children are seldom of a curative nature and many patients require corrective procedures, palliative procedures and reoperations reaching adolescence and adulthood [2]. Pulmonary insufficiency (PI) secondary to pulmonary conduit failure is the most common residual finding during mid- to long-term follow-up and the most common indication for re-operation [3, 4]. Previously considered a benign lesion, studies show that PI leads to a dilatation of the right ventricle [5] and an impaired right ventricular function [5, 6]. Eventually, PI leads to RV dilatation with poor exercise tolerance [7] and long-term morbidity with potential lethal complications such as arrhythmias and sudden death [8]. The golden standard treatment for PI in patients with corrected TOF is pulmonary valve replacement (PVR) with a bioprosthesis or a conduit [9]. However, the timing of PVR in conduit failure and secondary PI remains a subject of debate and the current ESC Guidelines for the Management of Grown-up Congenital Heart Disease and the 2008 ACC/AHA guidelines show that normalization of right ventricular size after re-intervention becomes unlikely as the end-diastolic volume index exceeds 160 mL/m^2^ [10, 11]. The aim of this study was to evaluate the potential for RV reverse remodelling after PVR using cardiac magnetic resonance imaging (cMRI), in adults with corrected TOF and severe late PI.

## Materials and methods

### Study design

The present study was a prospective, non-randomized observational study in which patients with previous correction of TOF with severe PI accepted for PVR were included. Patients were evaluated with cMRI preoperatively and re-evaluated 6 to 12 months postoperatively, according to a study protocol. Permission to proceed with this study was granted by the institutional Ethics Review Board, Lund, Sweden and individual consent was obtained from all patients enrolled.

### Indication for PVR

The current indications for PVR for severe PI in patients with corrected TOF according to the most recent guidelines [10, 11] are based on the presence of symptoms (Class I). In asymptomatic patients, the indications are restricted to the following situations: decrease in exercise tolerance according to objective tests; deterioration in RV function and RV dilatation; presence of sustained atrial and/or ventricular arrhythmias; tricuspid regurgitation (at least moderate); and RV outflow tract obstruction (Class IIa). In the current study, PVR was indicated due to the presence of severe PI and RV dilatation in all cases but two. In the first case progressive symptoms of fatigue and dyspnoea (NYHA II) in addition to PI and RV dilatation contributed to the interventional decision. In the second case, PVR was indicated on progressive symptoms alone.

### Surgical technique

The RVOT Elan™ (Vascutek, Renfrewshire, UK) conduit (*n* = 5) [12] or the St Jude Epic mitral (St Jude™ Medical, Inc., St Paul, Minn, US.) bioprosthetic valve (*n* = 5), was implanted by one expert surgeon in adults with congenital heart disease. All implantations were performed on cardiopulmonary bypass support and moderate hypothermia. The majority of the procedures (*n* = 8) were performed without cardioplegic arrest. When necessary, myocardial protection was achieved with cold blood anterograde cardioplegia. Valve sizing was based on preoperative magnetic resonance imaging as well as intraoperative assessment of the RV outflow tract. Adequate function of the prosthesis was assessed by intraoperative transoesophageal echocardiography immediately after weaning from cardiopulmonary bypass. Postoperative anticoagulation consisted of acetylsalicylic acid (75 mg) three months postoperatively and low-molecular-weight heparin administered during the immobilization phase in the early postoperative period.

### Cardiac magnetic resonance imaging

Cardiac magnetic resonance imaging was the primary investigation technique for evaluation of RV hemodynamics [13–15]. Patients were examined in the supine position using retrospective ECG triggering with a 1.5 T Philips Achieva scanner (Philips Healthcare, Best, The Netherlands) or 1.5 T MAGNETOM Aera (Siemens Healthcare GmbH, Erlangen, Germany). Steady-state free precession cine images covering the entire heart was acquired. Typical imaging parameters for Philips scanner were TR/TE: 2.8/1.4, flip angle: 60°, slice thickness: 8 mm, no gap, reconstructed spatial resolution: 1.4 × 1.4 × 8 mm3, acquired temporal resolution 47 ms and for the Siemens scanner TR/TE: 2.7/1.2, flip angle: 70°, slice thickness: 8 mm, no gap, reconstructed spatial resolution: 1.2 × 1.2 × 8 mm3, acquired temporal resolution 43 ms. 2D through-plane flow measurements were performed in the ascending aorta and pulmonary artery to measure the effective stroke volume (SV) and pulmonary regurgitation. The freely available software Segment (http://segment.heiberg.se) was used for all image analysis [16].

### Follow up

Follow-up was performed in February 2017 and was 100% complete for survival (mean 37 ± 12 months, interquartile range 26–48). Follow-up with cMRI was performed after a mean of 10 ± 5 months postoperatively and was 100% complete.

### Definitions

Mean indexed RV end-diastolic volume (RVEDVI) was considered normal if ≤99 ml/ m^2^, RV end-systolic volume (RVESVI) was considered normal if ≤41 ml/m^2^, and mean indexed RV stroke volume (RVSVI) was considered normal if ≤65 [17]. *Postoperative bleeding* was defined as bleeding leading to exploration; postoperative *heart failure* was defined as the need for postoperative inotropic support (norepinephrine and/or dobutamine infusion) for more than 24 h; *acute myocardial infarction* was diagnosed by electrocardiography (ECG) (new permanent Q-wave), echocardiogram (regional wall movement abnormalities) and cardiac enzymes (aspartate aminotransferase (AST) > 2 μkat/L or creatine kinase myocardial specific isoform (CK-MB) > 50 μg/L); *postoperative atrial fibrillation* (AF) was defined as one or recurrent episodes of supraventricular tachyarrhythmia after cardiac surgery, monitored by continuous telemetry ECG or with an ECG recording using atrial epicardial leads. Early mortality was defined as all-cause mortality within 30 days of surgery.

### Statistics

Descriptive data are presented as number (%) or mean ± SD. All data were indexed for body surface area. The two-tailed paired Student’s t-test was used to compare the intra-individual pre-operative and postoperative data. In addition, Wilcoxon Rank test was used, compensating for lack of normal distribution. A *p*-value less than 0.05 was considered statistically significant. Statistical analyses were performed with the statistical software package SPSS (Version 21.0, IBM, Armonk, NY).

## Results

### Patient population

Patient characteristics are presented in Table 1. Mean age at the initial corrective procedure for TOF was 6 ± 9 years (median 1.8 years; range 0.3–29 years). Four patients underwent a palliative procedure (Blalock-Taussig shunt) before correction. Four patients had additional interventions post initial correction for TOF – three had pulmonary artery homograft implantation and one had a left pulmonary artery stent. One patient had undergone a percutaneous attempt of pulmonary valve replacement. However, the procedure was unsuccessful and conventional PVR was required.

**Table 1:** Baseline demographics (*n* = 10)

Age (years)	32 ± 12
Sex (female)	4 (40)
Body surface area (m^2^)	1.9 ± 0.2
Body mass index (kg/m^2^)	26 ± 5.5
Any cardiac medication	1 (10)
Diabetes mellitus	0
Hypertension	0
Current smoker	0
No of previous interventions	1.5 ± 0.5
History of arrythmia	0
NYHA class	
I	8 (80)
II	2 (20)
Laboratory values	
S-creatinine (μmol/L)	64 ± 16
B-Haemoglobin (g/L)	144 ± 11
Surgical correction for TOF	6 ± 9
Age at primary correction (years)	
Previous palliative shunt	4 (40)
BT-shunt	

Values are expressed as number (%) or mean ± SD (median, range); NYHA, New York Heart Association; eGFR, estimated Glomerular filtration rate; TOF, Tetralogy of Fallot; BT-shunt, Blalock-Taussig shunt

### Intra- and postoperative data

There was no intraoperative or early mortality. The mean time interval since the last intervention was 20 ± 14 years. The mean pulmonary prosthetic valve size was 25 ± 1.5 mm. Intraoperative data and postoperative outcome are demonstrated in Table 2. Postoperative complications occurred in 3 (30%) patients, including sternal infection (*n* = 1), supraventricular tachycardia (*n* = 1), and low cardiac output syndrome (*n* = 1). One patient presented with dyspnoea and flank pain three weeks postoperatively and was diagnosed with pulmonary embolism. The patient was subsequently treated with Warfarin and the medical work up revealed a heterozygote carrier for the prothrombin gene mutation. The mean length of postoperative hospitalization was 10 ± 4 days. All patients were alive at the time of follow-up.

**Table 2:** Intra- and postoperative characteristics

Type of conduit	
Epic bioprosthetic mitral valve conduit	5 (50)
RVOT Elan bioprosthetic heart valve conduit	5 (50)
Pulmonary valve replacement	
Perfusion time (min)	85 ± 20
Aortic cross-clamping (min)	7 ± 15
Lowest temperature (°C)	35 ± 1
Previous interventions	
PA-homograft	3 (30)
LPA-stent	1 (10)
Palliative procedures	
BT-shunt	4 (40)
Time since last intervention (years)	20 ± 14
Time since total correction	27 ± 11
Complications	1 (10)
Arrhythmia	
New onset SVT	1 (10)
Infection	1 (10)
Pre-sternal	0
Heart failure	1 (10)
Myocardial infarction	
Other complications	10 ± 4
Postoperative hospitalization (days)	
Duration ICU stay (days)	1 ± 0

Values are expressed as number (%) or mean ± SD (median, range); PA-homograft, pulmonary artery homograft valve; LPA-stent, placement of stent in the left pulmonary artery; *SVT* supra-ventricular tachycardia, *ICU* Intensive care unit

### Hemodynamic data

**Fig. 1:**
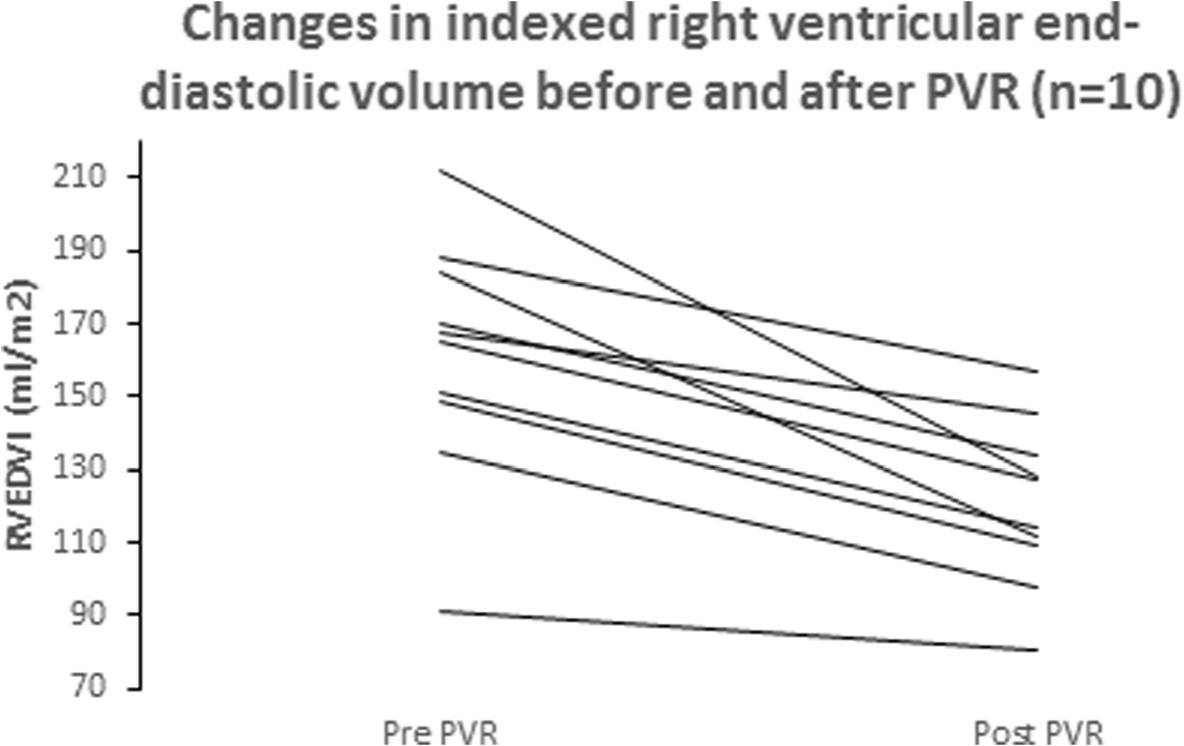
Changes in indexed right ventricular end-diastolic volume and after PVR

Pre- and postoperative cMRI data are in presented in Table 3. Preoperatively, all patients had at least a moderate PI. The PI fraction decreased from 49% ± 11 to 1% ± 1 postoperatively, *p* < 0.001. The preoperative RVEDVI was reduced from 161 ± 33 ml/m^2^ (range, 91 to 212 ml/m^2^) to 120 ± 23 ml/m^2^ (range, 80 to 157 ml/m^2^) postoperatively, *p* < 0.001 (Fig. 1). The preoperative RVESVI was reduced from 89 ± 25 ml/m^2^ (range, 28 to 125 ml/m^2^) to 71 ± 21 ml/m^2^ (range, 29 to 106 ml/m^2^) postoperatively, *p* = 0.036. The pre- and postoperative RVEDVI for each patient is shown in Fig. 1. After PVR, the RVEF did not change significantly (46% versus 42%, *p* = 0.337). The preoperative RVSVI was reduced from 72 ± 20 ml/m^2^ (range 43 to 107 ml/m^2^) to 50 ± 6 ml/m^2^ (range 37 to 57 ml/m^2^) postoperatively, *p* = 0.002. The preoperative mean net flow in the main pulmonary artery (MPA) was 5100 ± 1500 ml/min (range, 3300 to 7700 ml/min) with an increase to mean 6100 ± 800 ml/min (range, 5400 to 8000 ml/min) postoperatively, *p* = 0.120.

**Table 3:** Pre- and postoperative hemodynamic data (cMRI)

Variables (*n* = 10)	Before PVR	After PVR	*Students t-test*	*Wilcoxon Signed Rank Test*
RVEDV (ml)	309 ± 85	231 ± 45	0.001	0.005
RVEDVI (ml/m^2^)	161 ± 33	120 ± 23	< 0.001	0.005
RVESV (ml)	171 ± 58	136 ± 40	0.043	0.047
RVESVI (ml/m^2^)	89 ± 25	71 ± 21	0.036	0.047
RVSV (ml)	138 ± 45	95 ± 13	0.006	0.005
RVSVI (ml/m^2^)	72 ± 20	50 ± 6	0.002	0.005
RVEF (%)	46 ± 11	42 ± 9	0.337	0.373
LVEDV (ml)	149 ± 22	171 ± 28	0.002	0.007
LVEDVI (ml/m^2^)	79 ± 10	90 ± 15	0.012	0.015
LVESV (ml)	68 ± 17	76 ± 20	0.213	0.236
LVESVI (ml/m^2^)	36 ± 8	40 ± 11	0.271	0.284
LVSV (ml)	81 ± 12	95 ± 12	< 0.001	0.005
LVSVI (ml/m^2^)	43 ± 7	50 ± 6	0.001	0.005
LVEF (%)	55 ± 7	56 ± 6	0.656	0.593
PI (%)	49 ± 11	1 ± 1	< 0.001	NA
MPA netflow (mL/min)	5100 ± 1500	6100 ± 800	0.120	0.139

Values are expressed as number (%) or mean ± SD (median, range); EF, ejection fraction; RVEDV, right ventricular end-diastolic volume; RVESV, right ventricular end-systolic volume; RVSV, right ventricular stroke volume; LVEDV, left ventricular end-diastolic volume; LVESV, left ventricular end-systolic volume; LVSV, left ventricular stroke volume; PI, pulmonary insufficiency; MPA, main pulmonary artery

The preoperative mean indexed left ventricular stroke volume (LVSVI) increased from 43 ± 7 ml/m^2^ (range, 33 to 56 ml/m^2^) to 50 ± 6 ml/m^2^ (range, 40 to 58 ml/m^2^) postoperatively, *p* = 0.001. The preoperative mean indexed left ventricular end-systolic volume (LVESVI) increased from 36 ± 8 ml/m^2^ (range, 26 to 48 ml/m^2^) to 40 ± 11 ml/m^2^ (range, 21 to 58 ml/m^2^) postoperatively, *p* = 0.271. The preoperative mean indexed left ventricular end-diastolic volume (LVEDVI) increased from 79 ± 10 ml/m^2^ (range, 77 to 97 ml/m^2^) to 90 ± 15 ml/m^2^ (range, 61 to 108 ml/m^2^) postoperatively, *p* = 0.012.

## Discussion

Data in the present study indicates that even though the preoperative mean RVEDVI exceeded 160 mL/m^2^ in the majority of the cases, there was a significant reverse remodelling with a reduction in right ventricular end-diastolic volume 6–12 months postoperatively. However, the RVEDVI did not reach normal values postoperatively in any case but one.

Optimal timing of PVR due to severe PI in TOF is still a subject of debate as beneficial effects of PVR have to be weighed against the risk for repeat PVR. Performing PVR early during follow-up could be associated with an improved RV remodelling [18–21], but it may increase the number of surgical interventions during the patient’s lifetime and increasing the accumulated risk of procedure-related complications. However, Vliegan et al. has previously shown that RV volumes were significantly reduced after PVR in adult patients with corrected TOF [22] and attempts have been made to find a cutoff where the RV does not normalize postoperatively in order to facilitate the decision for optimal timing of intervention. Cut-off values for indexed RV end-diastolic volume (RVEDV) between 150 and 170 ml/m^2^ have been proposed as an indication for PVR in asymptomatic adults with congenital heart disease with previously corrected TOF. Therrian et al. has previously reported that the RV does not reach normal volumes following surgery if the preoperative RVEDVI exceeds 170 ml/m^2^ [23]. Similarly, in children a cut-off of 200 ml/m^2^ has been shown to predispose for an incomplete reverse remodelling [24]. According to the current guidelines [10, 11] normalization of RVEDVI becomes more unlikely when the preoperative RV volume exceeds the cut-off of 160 ml/m^2^. In the current study, the preoperative mean RVEDVI was 161 ± 33 ml/m^2^. The majority of the patients did not reach normal RVEDVI (≤ 99 ml/ m^2^) 6–12 months postoperatively as assessed by MRI. This incomplete pattern of reverse remodeling could also be shown for patients with RVEDVI lower than the cut-off of 160 ml/ m^2^. However, a significant reverse remodeling was present in all patients despite not reaching normal postoperative RV volumes. In addition, RVSVI decreased after surgery, reaching normalization. Data in the present study showing early RV reverse remodeling are consistent with those of previous studies [18, 25–28]. In a meta-analysis including 48 studies involving 3118 patients, Ferraz Cavalcanti et al. [25] showed similar favorable remodeling of the RV mostly between 1 and 3 years after PVR. No clear cutoff for preoperative RVEDVI that “guarantees” normal postoperative RV size has thus been identified. Furthermore, in a report by van Straten et al. RVEDVI decreased only marginally (2 ml/m^2^) from 7 to 18 months after PVR [29]. This is supported by, Hallbergsson et al. [21] showing that favorable RV reverse remodeling early after PVR erodes during long-term follow-up, with a gradual return toward dilatation and pre-PVR values of RV size and function up to 10 years postoperatively. This is probably associated with increasing PI and pulmonary stenosis related to deteriorating function of the implanted conduit and, in some patients, volume load from tricuspid regurgitation. Therefore, given that the negative consequences of moderate or even severe RV dilatation are questionable, focusing on preoperative RV function appears to be more appropriate.

In the present study, data indicated a positive change in right-to-left ventricular interaction after surgery. The RVEF did not change significantly, 46% versus 42% (*p* = 0.337) after PVR. However, the preoperative RVEF does not show the actual ejection fraction as approximately 50% of the stroke volume is regurgitated to the RV. This implies that the RVEF actually improves postoperatively when there is only negligible remaining PI. Furthermore, an increase in LVEDVI, LVESVI and left ventricular stroke volume could be observed postoperatively. Our findings are supported by Oosterhof et al. showing that RV dysfunction may negatively affect left ventricular (LV) function as RV volume overload due to PI correlated to lower RVEF and LVEF as well as higher BNP levels [30]. In our study, both RVEDVI and RVESVI decreased after surgery while net-flow in the main pulmonary artery increased indicating an enhanced RV output. The fact that LVEDVI, LVESVI and LV stroke volume increased after surgery while LVEF remained unchanged may reflect a more favourable right-to-left ventricular interaction with equal stroke volumes and a trend towards improvement of the LV function.

### Clinical implications

This was a prospective study with a consistent cMRI protocol with the following clinical implications. The improvement in RV size following PVR compared to pre PVR measurements assists clinical decisions regarding timing of valve or conduit replacement. However, no clear cutoff for preoperative RVEDVi that “guarantees” normalization of RV size post PVR could be identified. Moreover, given that previous data [21] show gradual RV enlargement and a decline in global RV systolic function toward pre-PVR values the importance of subsequent surveillance is highlighted. Follow-up should include quantitative assessment of ventricular size and function, bioprosthetic pulmonary valve function - preferably by cMRI. In addition, our results highlight the importance of improving strategies to maintain pulmonary valve competence over several decades. Specifically, given that the inevitable functional deterioration of all currently available bioprosthetic valves and conduits leads to worsening RV mechanics.

### Limitations

One limitation of this study is the lack of a control group. The relatively small number of patients results in a loss of statistical power, requiring validation using larger groups of patients. Even though our data have been validated previously in larger studies, these have been retrospective and heterogeneous in contrast to our prospective study.

## Conclusion

PVR leads to a favourable early reverse remodelling with a reduction in RV volumes and improved biventricular function. A significant reverse remodeling was present in all patients despite not reaching normal postoperative RV volumes.

## Abbreviations

AST: Aspartate aminotransferase
CK-MB: Creatine kinase myocardial specific isoform
cMRI: Cardiac magnetic resonance imaging
ECG: Electrocardiography
LV: Left ventricular
LVEDVI: Mean indexed left ventricular end-diastolic volume
LVESVI: Mean indexed left ventricular end-systolic volume
LVSVI: Mean indexed left ventricular stroke volume
MPA: Mean net flow in the main pulmonary artery
PI: Pulmonary insufficiency
PVR: Pulmonary valve replacement
RV: Right ventricular
RVEDVI: Mean indexed right ventricular end-diastolic volume
RVESVI: Mean indexed right ventricular end-systolic volume
RVSVI: Mean indexed right ventricular stroke volume
SV: Stroke volume
TOF: Tetralogy of Fallot
